# A high-efficiency CRISPR/Cas9 system for targeted mutagenesis in Cotton (*Gossypium hirsutum* L.)

**DOI:** 10.1038/srep43902

**Published:** 2017-03-03

**Authors:** Chao Li, Turgay Unver, Baohong Zhang

**Affiliations:** 1 Department of Biology, East Carolina University, Greenville, NC 27858, USA; 2 Izmir International Biomedicine and Genome Institute (iBG-izmir), Dokuz Eylul University, Balcova 35340, Izmir, Turkey

## Abstract

The complex allotetraploid genome is one of major challenges in cotton for repressing gene expression. Developing site-specific DNA mutation is the long-term dream for cotton breeding scientists. The clustered regularly interspaced short palindromic repeats/CRISPR-associated protein 9 (CRISPR/Cas9) system is emerging as a robust biotechnology for targeted-DNA mutation. In this study, two sgRNAs, GhMYB25-like-sgRNA1 and GhMYB25-like-sgRNA2, were designed in the identical genomic regions of *GhMYB25-like A* and *GhMYB25-like D*, which were encoded by cotton A subgenome and the D subgenome, respectively, was assembled to direct Cas9-mediated allotetraploid cotton genome editing. High proportion (14.2–21.4%) CRISPR/Cas9-induced specific truncation events, either from *GhMYB25-like A* DNA site or from *GhMYB25-like D* DNA site, were detected in 50% examined transgenic cotton through PCR amplification assay and sequencing analyses. Sequencing results also demonstrated that 100% and 98.8% mutation frequency were occurred on GhMYB25-like-sgRNA1 and GhMYB25-like-sgRNA2 target site respectively. The off-target effect was evaluated by sequencing two putative off-target sites, which have 3 and 1 mismatched nucleotides with GhMYB25-like-sgRNA1 and GhMYB25-like-sgRNA2, respectively; all the examined samples were not detected any off-target-caused mutation events. Thus, these results demonstrated that CRISPR/Cas9 is qualified for generating DNA level mutations on allotetraploid cotton genome with high-efficiency and high-specificity.

Cotton fiber and its derivative products play crucial roles in our daily life and the world economy, which had been estimated to directly determine the annual income of almost 100 million families from approximately 150 countries^1^. Its annually worldwide economic impact had been assessed at approximately US$500 billion^1^,^2^. The widely cultivated cotton cultivars are allotetraploid species, which consist of two set of subgenomes, “A subgenome” and “D subgenome”. Those two subgenomes cotton species reunited geographically by the transoceanic dispersal happened approximately 1–2 million years ago (MYA)^3^. This polyploidization confers many excellent properties on tetraploid cotton, including longer cotton fiber length and higher cotton fiber strength, which make it possible to cultivate modern spinnable cotton cultivars. However, the complex genome feature of allotetraploid cotton presents a new challenge for cotton genes functional analyses and the genetic improvement through transgenic approach. Previously, many cotton genes were identified to be implicated in cotton fibre development^4^,^5^,^6^,^7^,^8^,^9^,^10^,^11^,^12^,^13^,^14^,^15^,^16^, stress responsees^17^,^18^,^19^,^20^, and pathogen immune regulation through expressed sequence tag (EST)-based cDNA library coupled with conventional RNAi and gene overexpression strategies^21^,^22^,^23^,^24^. With the rapid development of high throughput deep sequencing technologies and bioinformatics, many diploid and allotetraploid cotton species were sequenced and assembled within recent years, including D-genome diploids cotton *Gossypium raimondii* (DD; 2n = 26)^25^,^26^, A-genome diploids *Gossypium arboreum* (AA; 2n = 26)^27^, ant the AtDt allotetraploid cotton species *Gossypium hirsutum* and *Gossypium barbadense*^28^,^29^,^30^. Those excellent contributions extremely facilitate gene identification of interest and subsequent vector construction for functional genes analyses and screening via ‘genotype-to-phenotype’ approach. Thus, the arsenal of cotton genomic manipulation urgently require to be updated to meet the demand for rapid and precise dissecting gene functional analyses.

Extensive studies have shown that high-frequent creating DNA double-strand breaks (DSBs) in desired nuclear DNA sites is a reliable approache to induce gene mutations^31^. DSBs can trigger two distinct endogenous DNA repair mechanisms, error-prone nonhomologous end joining (NHEJ) and homology-directed repair (HDR)^32^,^33^, respectively. For NHEJ-mediated repair, it simply bring the break ends together and rejoin them by DNA ligation without the guidance from homologous template. Therefore, NHEJ was considered as a “quick and dirty” approach to repair DSB-caused DNA breaks and frequently lead to indel mutations and loss of nucleotides at the repair sites^33^,^34^,^35^. In contrast to NHEJ, the completion of DHR depends on the homologous sequence as a guide template, which usually is its undamaged sister chromatid, and so produces more accurate repair than NHEJ^34^,^35^,^36^.

To artificially generate targeted DSBs in the genomic region of interest, several nucleases, including meganucleases (MN), zinc-finger nucleases (ZFN), transcription activator-like effector nucleases (TALEN), were engineered to catalyze site-specific cleavage through fusing a programmable, sequence-specific DNA-binding domain with a cleavage domain^37^,^38^,^39^,^40^,^41^,^42^,^43^,^44^.

The type II RNA-guided CRISPR/Cas9 system, which derived from the adaptive immunity mechanism of bacteria *Streptococcus pyogenes*, has been recently proven to be effective for targeted gene editing in a wildly range of organisms, including human cells, mice, zebrafish, bacteria, yeast, *Arabidopsis thaliana, Nicotiana benthamiana*, maize, wheat, populus, grape sorghum and rice^36^,^45^,^46^,^47^,^48^,^49^,^50^,^51^,^52^,^53^,^54^,^55^,^56^,^57^,^58^,^59^,^60^,^61^,^62^,^63^,^64^. The CRISPR/Cas9 system is composed of a Cas9 nuclease sequence and two noncoding RNA genes, a precursor CRISPR RNA (pre-crRNA) and a trans-activating crRNA (tracrRNA). By replacing those two RNA genes with an engineered single guide RNA (sgRNA), the sgRNA-Cas9 complex can specifically recognizes complementary DNA targets sequence that immediately upstream of a 5′-NGG or 5′-NAG protospacer adjacent motif (PAM) sequence through Watson-Crick base pairing, and then catalyzing a site-specific cleavage on the targeted DNA sequence 3–4 base pairs upstream of the PAM site. In this study, a set of CRISPR/Cas9 genome editing system was firstly proved to possess the feature of high-efficiency and high-specificity on allotetraploid cotton genome editing, which may extremely enhance cotton genomic study and application.

## Results

### Experimental design and Golden Gate assembly of sgRNAs

CRISPR/Cas9 technology is emerging as important genome manipulation techniques for precisely gene targeting and DNA editing. Given that the genome complexity of allotetraploid upland cotton, we sought to develop a high-efficient and time-saving CRISPR/Cas9 system for cotton research community. Based on optimizing maize-codon Cas9 protein and simplifying the assembly process of sgRNAs, Xing and colleagues (2014) validated the high efficiency and specificity of a set of CRISPR/Cas9 toolkit in model plant Arabidopsis^45^. Most importantly, the multiple-gene mutations could be transmitted to their progenies with the efficiency can reach up to 100%^45^.

**Figure 1:**
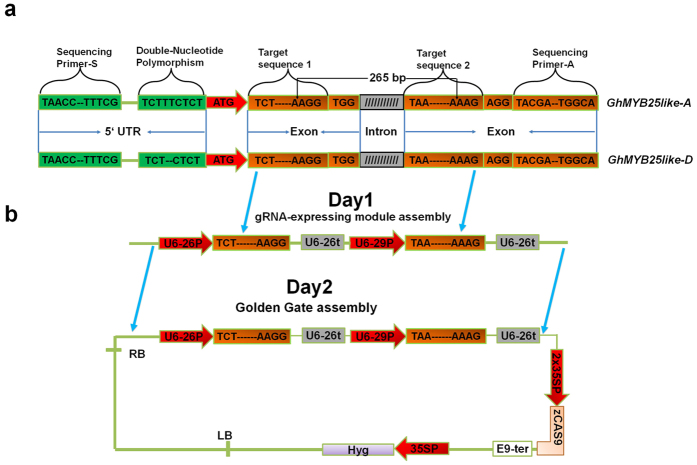
Overview of CRISPR–Cas9-guided genome editing in *GhMYB25-like A* and *GhMYB25-like D* genes. (**a**) Schematic description of selected DNA region of *GhMYB25-like A* and *GhMYB25-like D*. Two 23-bp 5′-N_20_NGG-3′ types of genomic DNA sequence are derived from the exons regions of *GhMYB25-like A* and *GhMYB25-like D*. The target sequences lying immediately upstream of the PAM sequence, TGG and AGG, were selected as guide sequence. The designed cleavage length is 265 bp. A pair of sequencing primer will be used for checking the effectiveness and efficiency of targeted DNA sites. And there are several single-nucleotide polymorphism and double-nucleotide polymorphism within sequencing region, which can be used to distinguish gene *GhMYB25-like A* and gene *GhMYB25-like D*. (**b**) Diagrams illustrating the procedure of vector construction. The upper panel is the premade gRNA-expressing modules. U6-26p and U6-29p are Arabidopsis U6 gene promoters, and U6-26t is Arabidopsis U6 gene terminators. The lower panel is the main functional component of this CRISPR/Cas9 binary vectors between the RB and LB. Both Cas9 protein and Hyg selectable markers are driven by 2X35 S promoter. *ZCas9* and Hyg are *Zea mays* codon-optimized Cas9 sequence and hygromycin respectively.

Based on screening bacterial artificial chromosomes (BACs) library, two *GhMYB25-like* cDNAs, referred to as *GhMYB25-like A* and *GhMYB25-like D*, were identified from upland cotton^6^. Although *GhMYB25-like A* and *GhMYB25-like D* are encoded by allotetraploid cotton A subgenome and the D subgenome respectively, they share a similar gene structure and highly conserved R2R3-binding domain^6^. Previous study show that *GhMYB25-like* play important roles in cotton fiber development^6^. Thus, *GhMYB25-like A* and *GhMYB25-like D*, which derived from A subgenome and the D subgenome, respectively, are optimum candidates for validating the effectiveness of CRISPR/Cas9 genome editing system. Through searching the DNA sequence of *GhMYB25-like A* and *GhMYB25-like* D, two 23-bp 5′-N_20_NGG-3′ types of genomic DNA sequence were chosen as target sites for designing CRISPR/Cas9 vectors (Fig. 1a).

By using PCR-based sgRNA(guide RNA) assembly system, two sgRNAs, GhMYB25-like-sgRNA1 and GhMYB25-like-sgRNA2, were rapidly introduced into sgRNA-expressing module with just one round of PCR reaction. As shown in Fig. 1b, the expressions of GhMYB25-like-sgRNA1 and GhMYB25-like-sgRNA2 were driven by Arabidopsis Pol III promoters, U6-26p and U6-29p, respectively. And each of GhMYB25-like-sgRNA1 and GhMYB25-like-sgRNA2 have their own terminators, U6-26t (Fig. 1b). The application of sgRNA-expressing module vectors facilitates the assembly process, and meanwhile guarantee the accuracy of those sgRNA expression cassettes. In my case, one day is sufficient to accomplish PCR amplification and PCR products purification. In the following step, the Type IIS restriction endonucleases (REases) *BsaI*, was employed to seamlessly integrate maize-codon optimized *Cas9* and two GhMYB25-like sgRNA-expressing cassettes (Fig. 1b), which could be finished within 6 hours.

### Gene transformation and evaluation of CRISPR/Cas9-mediated mutagenesis in cotton *GhMYB25-like A and GhMYB25-like D*

**Figure 2:**
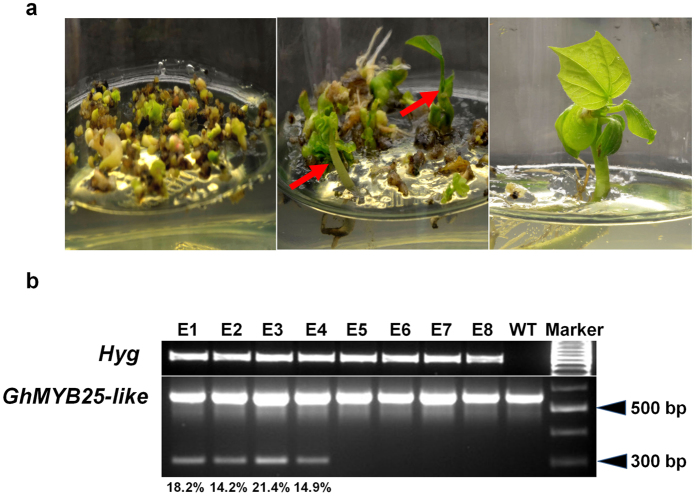
Cotton transformation and identification of positive plantlets for mutation analyses. (**a**) Regeneration of cotton *GhMYB25-like* transgenic plants. Sterilized cotton hypocotyl were excised into 5–8 mm segments for infecting with GhMYB25-like::CRISPR/Cas9-containing *A. tumefaciens* strain EHA105. Left panel is the hygromycin-resistant cotyledonary stage embryos generated from original hypocotyl segments, middle and right panel is part of regeneration plantlets. (**b**) Detection of CRISPR/Cas9-induced long DNA fragment deletions. The common PCR primers of *GhMYB25-like A* and *GhMYB25-like D* were synthesized for double-cleavage mutation analyses and genes cloning.

*Agrobacterium tumefaciens (A. tumefaciens*)-mediated cotton transformation and somatic embryogenesis were performed as described previously^23^,^65^,^66^. With several round of tissue subculture and antibiotic selection on selective medium, a lot of hygromycin-resistant cotyledon-stage embryos were generated from original explant (Left panel, Fig. 2a), excised cotton hypocotyl segments. Those antibiotic-resistant embryos continued to be cultured on hygromycin-containing medium and many plantlets were produced on selective medium (middle panel, Fig. 2a). Two plantlets, as shown by red arrow on Fig. 2a, from each independent transgenic event were sampled for DNA extraction and subsequent mutation analyses. To validate the exogenous T-DNA insertion in *GhMYB25-like* transgenic plantlets, DNAs extracted from twelve independent transgenic events were analyzed by PCR assay using gene specific primers for the hygromycin resistant gene. And eight DNA samples were detected the correct exogenous T-DNA insertion (Upper panel, Fig. 2b), which were referred to E1, E2, E3, E4, E5, E6, E6, E8, respectively.

To evaluate the potential genomic DNA deletion occurred on the designed *GhMYB25-like* genomic regions, a pair of primers, covering the similar genomic area of *GhMYB25-like A* and *GhMYB25-like D* (Fig. 1a), was synthesized to detect the truncated cleavage product. As shown in lower panel of Fig. 2b, a specific smaller band, which below the main PCR product and the size is around 300 bp, was found in samples E1, E2, E3, E4, whereas only one main PCR band was detected in samples E5, E6, E7, E8. Based on our original design, the size of the main PCR product is 572 bp, and the designed cleavage length is 265 bp (Fig. 1a). Thus, our PCR results demonstrated that the precise cleavage events most probably occurred in our designed genomic regions of *GhMYB25-like* genes.

**Figure 3:**
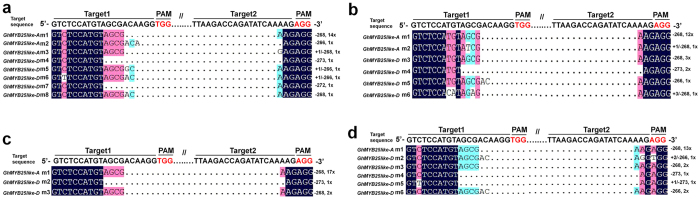
Validation of CRISPR/Cas9-induced long DNA fragment deletions at both *GhMYB25-like A* and D subgenomic sites. (**a**) to (**d**) Alignment of genomic sequences cloned from the truncated PCR products using E1, E2, E3, E4 DNA samples as templates.

To further validate whether the changed PCR size was derived from CRISPR/Cas9-caused genomic truncation, we randomly picked 20 positive colonies generated from smaller PCR products of each transgenic cotton sample E1/E2/E3/E4 for sequencing analyses. Those sequencing results demonstrated that all 80 smaller sequences were the truncated versions of *GhMYB25-like* genomic sequences, either from *GhMYB25-like A* DNA site or from *GhMYB25-like D* DNA site (Fig. 3). To quantify the proportion of double cleavage, signal intensity of each band was measured by using ImageJ software ( https://imagej.nih.gov/ij/download.html). The cleavage DNA length was mostly concentrate on −268bp, which account for 87% and 92% in *GhMYB25-like A* DNA site of E1 and E2 samples, 100% in *GhMYB25-like A* DNA site of E1 and E2 samples, 67% in *GhMYB25-like D* DNA site of E3 sample (Table 1). Taken all together, these results indicated that this set of CRISPR/Cas9 genome editing system have the potential to efficiently generate long DNA fragment deletions on the selected genomic region.

**Figure 4:**
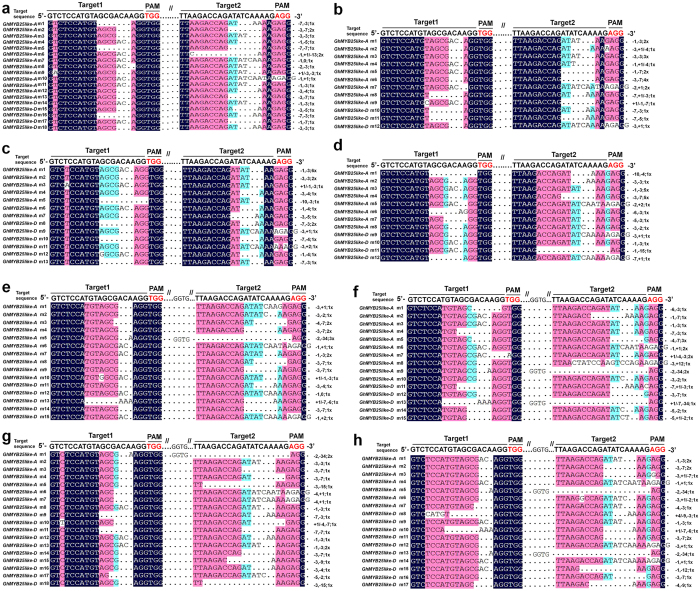
Sequencing analyses and validation of CRISPR/Cas9-induced NHEJ mutations at both *GhMYB25-like A* and D subgenomic sites. Sequence confirmation of nucleotide deletion and insertion mutations in cotton transgenic samples E1, E2, E3, E4, E5, E6, E7, and E8.

To investigate whether CRISPR/Cas9-mediated nucleotide insertion mutations and deletion events also precisely occurred in the main PCR products, 160 positive colonies, which cloned from the PCR products using E1, E2, E3, E4, E5, E6, E7, E8 DNA samples as templates, were randomly picked for sequencing analyses. In samples E1, E2, E3, E4, all the 159 examined Target1 and Target2 genomic sites precisely occurred genome editing events, except 1 DNA sites, which from E1 sample Target2, was not affected (Fig. 4). As shown in Table 2, most of the nucleotide insertion and deletion mutations were −1bp/−3bp/−7bp nucleotide deletion mutations and +1 bp insertion mutation.

Similarly, high proportion nucleotide insertion and deletion events were detected in samples E5, E6, E7, and E8 (Fig. 4). Except 1 DNA sites, which from E5 Target 2, still keep its wild type DNA sequence, all the rest of 159 examined genomic sites were detected nucleotide insertion or deletion mutation events (Fig. 4). As shown in Table 2, the nucleotide insertion and deletion mutations were mostly concentrate on −1bp/−2bp/−3bp/−7bp deletion mutations and +1 bp insertion mutation.

Thus, those results suggested that both GhMYB25-like-sgRNA1 and GhMYB25-like-sgRNA2 effectively and precisely guided cas9-mediated genome cleavage. Given that the high-efficient effect on both *GhMYB25-like A* and *GhMYB25-like D* genome sequence, this set of CRISPR/Cas9 genome editing system have the potential to generate DNA level knockout mutations on complex allotetraploid cotton genome.

Among the genome knockout transgenic events, mosaicism was observed in each transgenic event. Mosaicism sometimes may disturb later phenotypic analysis. Given that the double-cleavage DNA length was mostly on −268bp and the majority of small nucleotide mutations are −1bp/−3bp/−7bp nucleotide deletions, we infer that most but not all of the targeted genome editing events may occur in the transformed single cell stage. This can be eliminated during later stage of selection.

### Off-target analyses

**Figure 5:**
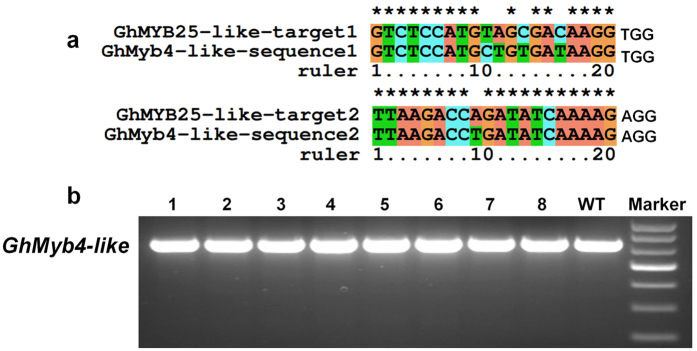
Off-targets analyses of two potential off-target sites. (**a**) Alignment of two putative off-target sites derived from *GhMYB4-like* genomic sequence. (**b**) Assessment of possible off-target-caused double-cleavage mutation.

To evaluate the possibility of off-target effect, two putative off-target sequences, which derived from *GhMYB4-like* genomic sequence and have three and one mismatched nucleotides with GhMYB25-like-sgRNA1 and GhMYB25-like-sgRNA2, respectively (Fig. 5a), were employed to analyzing potential off-target events. Unlike the result in Fig. 2B, the truncate smaller band was not detected by PCR amplifying the predicted GhMYB4-like genomic region (Fig. 5b). To further exam the potential involvement of small nucleotide insertion and deletion mutations, PCR product amplified from DNA sample E3, which have the highest proportion (21.4%) of double cleavage and 100% small nucleotide deletion mutation (Table 1 and Fig. 3c), was cloned for sequencing analyses. As shown in Table 3, both of the putative off target sites, GhMYB4-like-sequence1 and GhMYB4-like-sequence2, were not detected any occurrence of mutation events. Those data suggested that this set of CRISPR/Cas9 genome editing system have high specificity.

## Discussion

Cotton acts as one of the world’s major staple crop, contributing to approximately US$500 billion worldwide economic impact annually. Although the allotetraploid genome feature of upland cotton, which bring the challenges for cotton genetic improvement, cotton investigators never stop updating their biotechnology arsenal to more effectively and accurately dissect cotton genes functions, such as RNAi technology^23^, virus-induced gene silencing (VIGS) technology^23^,^67^, activation tagging technology^68^, constitutively or spatiotemporal gene expression technology^69^. With the revolutionary achievements in the cotton whole-genome sequencing and assembly, cotton research community urgent need a set of high-efficient and time-saving CRISPR/Cas9 system for cotton functional genome studies and the subsequent application.

To exam the qualification for effective CRISPR/Cas9-caused genomic editing in allotetraploid cotton genome, two sgRNAs (GhMYB25-like-sgRNA1 and GhMYB25-like-sgRNA2), designed in the identical genomic regions of A subgenome gene *GhMYB25-like A* and D subgenome gene *GhMYB25-like D*^5^,^6^, were employed to examine the efficacy of allotetraploid cotton genome editing. Through *A. tumefaciens*-mediated cotton transformation and antibiotic selection, eight independent positive transgenic plantlets samples, namely E1, E2, E3, E4, E5, E6, E6, E8 (Fig. 2B), were obtained for further analyzing the efficacy of the CRISPR/Cas9 system. Surprisingly, high proportion plausible cleavage products, which account for 18.2%, 14.2%, 21.4%, and 14.9% of the total PCR products produced from samples E1, E2, E3, and E4 DNA template (Fig. 2B), respectively, were detected directly through normal PCR amplification. Those potential double-cleavage events were further confirmed by sequencing analyses of those smaller PCR product, which showed that all of the 160 examined samples appeared to be derived from CRISPR/Cas9-triggered truncation events, either from truncated *GhMYB25-like A* genome region or from truncated *GhMYB25-like D* genome region (Table 1 and Fig. 3). The long genomic fragment deletions require high Cas9-sgRNAs-complex activity to ensure two designed cleavage sites be efficiently recognized and cleaved^70^,^71^. In addition, the accurate and high-efficient feature of this CRISPR/Cas9 system for long DNA fragment deletions provides the possibility to effectively replace undesired genomic area by introducing any desired engineered sequence fragment through endogenous HDR DNA repair mechanism^72^.

Current studies have demonstrated that most of DSBs are repaired by NHEJ-mediated repair mechanism and cause several nucleotide insertion mutations and deletion mutations^48^,^49^,^51^,^71^,^72^,^73^. Consistence with those discoveries, one main PCR product band, whose PCR sizes were very similar with their WT control lane, was detected in E1, E2, E3, E4, E5, E6, E7 and E8 DNA samples. However, sequencing analyses demonstrated that high proportion of nucleotide insertion and deletion occurred on predicted DNA cutting sites. Except two sites, which from E1 sample Target2 and E2 sample Target2, respectively, all the 318 examined sites exhibited mutations events (Fig. 4). Statistic data suggested that the majority of mutations detected in *GhMYB25-like A* and gene *GhMYB25-like D* genome were −1bp/−3bp/−7bp nucleotide deletions and +1 bp nucleotide insertion (Table 2). To sum up, these data demonstrated that this CRISPR/Cas9 genome editing system may be qualified for high-efficient generating DNA level mutations on allotetraploid cotton genome.

Off-target effect is a crucial factor for the application of CRISPR/Cas9 system. In our study, all the examined putative off-target sequences were completely match their original wild type genomic DNA sequence (Table 3), even though the second putative off-target sites only have 1 mismatched nucleotides with GhMYB25-like-sgRNA2 (Fig. 5). Several researches demonstrated that CRISPR/Cas9 system-caused off-target effect varies with different organisms and always very low in plants species^49^,^74^,^75^. Thus, this set of CRISPR/Cas9 system may have high-specificity in cotton genome editing.

**Figure 6:**
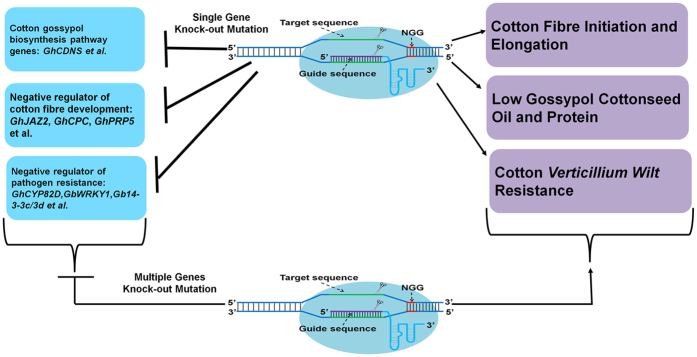
Prospect of the application of this set of CRISPR/Cas9 genome editing system. Cotton fibre and cottonseed oil/protein are two major agronomic traits directly related to cotton economic value. *Verticillium wilt* is the main bottleneck hampering cotton fibre production and quality in cotton field. Through the different sgRNA-expression modules, single or multiple sgRNAs can be easily assembled for the specific or integrated improvement of cotton fibre quality, gossypol toxicity of cottonseed and *Verticillium wilt* resistance.

Cotton is the most major source of nature textile fibre, and its cottonseed is emerging as one of the most impotent renewable resources of plant oil and plant protein. In addition, cotton *Verticillium wilt* is the most destructive disease, which annually cause 250–310 million US dollars economic losses in China^76^. Given that the high-efficiency, high-specificity, and high proportion of germline transmission of this CRISPR/Cas9 system^45^, we may have the expectation that it have the potential to substitute current mainstream RNAi vectors for generating DNA level knockout mutation in cotton genome. As shown in (Fig. 6), many candidate genes regarding to cotton gossypol biosynthesis, negative regulator of cotton fibre development and *Verticillium wilt* resistance, can be selected as target genes for genetic improvement of cotton agronomic traits. Recently, CRISPR/Cas9 have been modified to generate site-specific transcription activation/repression and targeted DNA methylation/demethylation by fusing different engineered enzymes^71^,^77^,^78^. However, the key first step for those modifications is the efficient and specific guidance of those engineered enzymes. Thus, this set of CRISPR/Cas9 system probably also have the potential to modify for inducing site-specific methylation/demethylation and transcription activation/repression, which will further facilitate cotton gene functional researches and applications.

## Methods and Materials

### Plant Material and Growth

Cotton (*Gossypium hirsutum* L.) cv ‘YZ1’ were used in this study. The plants were grown in the East Carolina University Greenhouse or growth chambers with a 16-h-day/8-h-night cycle at 28 °C.

### sgRNAs Design and Golden Gate Assembly of CRISPR/Cas9 System

To strict evaluate the efficacy and specificity of the CRISPR/Cas9 vectors, the sgRNAs design basically need to meet three standards: firstly, these sgRNAs target sites can be used to test the genome mutation efficacy on both *G. hirsutum* cotton A subgenome and D subgenome; secondly, several single-nucleotide polymorphism and double-nucleotide polymorphism near these two sgRNAs target sites can be used for distinguishing the identity of *GhMYB25-like* genes; thirdly, there are two highly similar sequences (have 1–3 mismatched nucleotides) with the designed sgRNAs can be used to estimate the off-target effect. Through comparing previously reported gene information and searching the NIBC cotton database, two sgRNAs, GhMYB25-like-sgRNA1 and GhMYB25-like-sgRNA2, which from *GhMYB25-like A* and *GhMYB25-like D* genomic sequences encoded by allotetraploid cotton A subgenome and the D subgenome respectively^5^,^6^, were designed for the assembly of sgRNA-expression module. The GhMYB25-like-sgRNA1 and GhMYB25-like-sgRNA2 expression module was assembled by direct PCR amplification using pCBC-DT1T2 template as described previously^45^. And then this PCR product was purified for the Golden Gate assembly of Cas9 expression module through the Type IIS restriction endonucleases (REases) BsaI reaction (R3535L, New England Biolabs).

### Cotton Transformation

The assembled CRISPR/Cas9 vector was then transformed into *Agrobacterium tumefaciens* (strain EHA105). And the *A. tumefaciens*-mediated cotton *G. hirsutum* ‘YZ1′ was performed as previous reports^65^,^66^,^79^.

### Genomic DNA Extraction and Mutations Analyses

The procedure of total genomic DNA isolation was followed the manual of Plant Genomic DNA Kit (TIANGEN, China). The specific primers designed from hygromycin resistance gene sequence were used for the identification of exogenous T-DNA insertion, and the primers sequence are Hyb-S2: TCGTTATGTTTATCGGCACTTTG, Hyb-A2: TATTGGGAATCCCCGAACAT. The common PCR primers of two *GhMYB25-like* genes were synthesized for double-cleavage mutation analyses and genes cloning, and the primers sequence are *GhMYB25-like*-S: TAACCAATTCTACCCACATTTTCG, *GhMYB25-like*-A: TGCCACTTTATCGGTTGTCGTA. PCR products were cloned by TA cloning reaction. And the positive colonies were randomly selected for sequencing (Genomics Core Facility, East Carolina University, NC). The sequences were analyzed and aligned through NCBI database and DNAman software.

### Off-target analyses

We searched all potential off-target sites in cotton genome. Two putative off-target sites, namely GhMYB4-like sequence1 and GhMYB4-like sequence2, were only discovered from *GhMyb4-like* (LOC107894856) genome sequence, which had three or less mismatches. GhMYB4-like sequence1 and GhMYB4-like sequence2 have 3 and 1 mismatched nucleotides with the designed GhMYB25-like-sgRNA1 and GhMYB25-like-sgRNA2, respectively (Fig. 5a). To detect the potential double-cleavage event, a pair of primers, covering the putative *GhMYB4-like* cleavage genomic area, was designed for PCR amplification and sequencing analyses. The PCR primers are GhMyb4-like-S: TTCCCTTGCTTTCAACGCTC, GhMyb4-like-A: GTTTTAGGCTTCTGCGTCACG. The sequencing analyses are same as mention above.

## Additional Information

**How to cite this article:** Li, C. *et al*. A high-efficiency CRISPR/Cas9 system for targeted mutagenesis in Cotton (*Gossypium hirsutum L.*). *Sci. Rep.* **7**, 43902; doi: 10.1038/srep43902 (2017).

**Publisher's note:** Springer Nature remains neutral with regard to jurisdictional claims in published maps and institutional affiliations.

## Figures and Tables

**Table 1:** Percentage of different CRISPR/Cas9-caused truncated events.

Sample	Gene	Rate of different nucleotide insertion (+) and deletion (−) (%)								
		−266	−268	−272	−273	+1/−273	+1/−266	+2/−266	+1/−268	+3/−268
E1	*GhMYB25like-A*	7	87						7	
	*GhMYB25like-D*		20	20	20		40			
E2	*GhMYB25like-A*		92						8	
	*GhMYB25like-D*	14	43		29					14
E3	*GhMYB25like-A*		100							
	*GhMYB25like-D*		67		33					
E4	*GhMYB25like-A*		100							
	*GhMYB25like-D*	29	29		14	14		14		

**Table 2:** Rate of CRISPR/Cas9-caused small nucleotide insertion (+) and deletion (−) events in cotton transgenic samples.

Sample	Target sequence	Gene	Rate of different nucleotide insertion (+) and deletion (−) (%)																						
			0	+1	−1	+1/−1	+1/−2	+1/−3	+1/−4	+1/−7	−2	++2	+2/−7	−3	−4	+4/−9	−5	−6	−7	−9	−10	−12	+12	−15	−34
E1	Target 1	*GhMYB25like-A*			40			7			7			33					13						
		*GhMYB25like-D*			40									40					20						
	Target 2	*GhMYB25like-A*	7	7	33										7		7		27		13				
		*GhMYB25like-D*												60			20		20						
E2	Target 1	*GhMYB25like-A*			29	6					12			47					6						
		*GhMYB25like-D*												33					67						
	Target 2	*GhMYB25like-A*		12					6					35	6				41						
		*GhMYB25like-D*		33										33	33										
E3	Target 1	*GhMYB25like-A*			54	8								31					8						
		*GhMYB25like-D*			14									29					57						
	Target 2	*GhMYB25like-A*			8									69	15		8								
		*GhMYB25like-D*		14								14		43	29										
E4	Target 1	*GhMYB25like-A*			31						6			44			6	6			6				
		*GhMYB25like-D*			50									25					25						
	Target 2	*GhMYB25like-A*		6							6			50	6				31						
		*GhMYB25like-D*		50										25										25	
E5	Target 1	*GhMYB25like-A*			20	7					20			40	13										
		*GhMYB25like-D*			40									40					20						
	Target 2	*GhMYB25like-A*		13							7			20					40						20
		*GhMYB25like-D*	20	20											20			20	20						
E6	Target 1	*GhMYB25like-A*			27						13			13	33			7	7						
		*GhMYB25like-D*												20			40		40						
	Target 2	*GhMYB25like-A*		13							7			33					27				7		13
		*GhMYB25like-D*				20		20			20								20						20
E7	Target 1	*GhMYB25like-A*			11						22			44	22										
		*GhMYB25like-D*			27				9					36			9		18						
	Target 2	*GhMYB25like-A*		22										22					22					11	22
		*GhMYB25like-D*									9			36	9				27	9				9	
E8	Target 1	*GhMYB25like-A*			33						11			44	11										
		*GhMYB25like-D*			27						9		9	36	9	9									
	Target 2	*GhMYB25like-A*		11			11			11				33					22						11
		*GhMYB25like-D*		18										18				9	27	9		9			9

**Table 3:** Mutation Analyses in two potential off-target sites.

sgRNA name	Putative off-target sequence	No. of mismatched nucleotides	No. of examined events	No. of mismatched events
GhMYB25-like-sgRNA1	GhMYB4-like-sequence 1	3	29	0
GhMYB25-like-sgRNA2	GhMYB4-like-sequence 2	1	29	0
